# The Kenyan assistive technology ecosystem: a network analysis

**DOI:** 10.1080/16549716.2024.2302208

**Published:** 2024-01-15

**Authors:** Emma M. Smith, Stephanie Huff, Rose Bukania, Bernard Chiira, Catherine Holloway, Malcolm MacLachlan

**Affiliations:** a Assisting Living and Learning Institute, Maynooth University, Maynooth, Ireland; b Ministry of Labour and Social Protection, Government of Kenya, Nairobi, Kenya; c Global Disability Innovation Hub, University College London, Nairobi, Kenya; d Global Disability Innovation Hub, University College London, London, UK

**Keywords:** Assistive products, disability, network analysis, self-help devices, kenya

## Abstract

**Background:**

Assistive technology is central to the realization of the rights of persons with disabilities. However, there remains limited access to assistive technology throughout much of the world, with particularly poor access in lower- and middle-income countries. Evaluating stakeholder engagement in assistive technology networks has been used as a successful strategy to understand and address gaps in the assistive technology ecosystem.

**Objective:**

The objective of this research was to provide an overview of the Kenyan Assistive Technology Ecosystem, including available assistive products and related services, and an understanding of the nature and strength of relationships between stakeholders

**Methods:**

In this study, we employed an online qualitative stakeholder survey (2021) with representatives of organizations involved in assistive technology in Kenya.

**Results:**

The assistive technology network in Kenya is distributed, with Government Ministries and Agencies and Organizations of persons with disabilities central to the network. The strength of relationships is concentrated on awareness and communication, with fewer organizations actively collaborating. Innovation training organizations are not yet well integrated into the network.

**Conclusions:**

Improving access to assistive technology in Kenya will benefit from greater collaboration amongst all assistive technology stakeholders.

## Introduction

The prevalence of disability in Kenya is estimated to be 2.2%, or nearly 1 million people [1]. Recent national census data indicates 1.9% of men and 2.5% of women have a disability in Kenya, with nearly half of all reported disabilities being mobility related (i.e. difficulty moving around the environment), followed by difficulties seeing and cognition [1]. In comparison to the 15% estimate of disability globally, the prevalence rate in Kenya is markedly lower and can be assumed to be underreported due to differences in the analysis and threshold used by the Kenya National Bureau of Statistics in its analysis, as well as the pervasive sociocultural stigma associated with disability [2], among other reporting barriers [3].

Individuals who experience one or more disabilities often rely on assistive technology to promote independence, overall health, and well-being. Assistive technology (AT) refers to ‘the systems and services related to the delivery of assistive products’, whereby assistive products (APs), such as mobility devices (e.g. wheelchairs, walkers, crutches) or communication aids (e.g. communication boards, text-to-speech communication devices), enable people with disabilities to maintain or improve function [4]. Assistive Technology-related services (AT-related services) refers to ‘the provision of information, training and assessment services, and follow up support’ [4].

However, assistive technology enables more than function: it is necessary to promote the health, well-being, social inclusion, and human rights of people with disabilities [4]. All people with disabilities have the right to available and affordable assistive technology [5], and in turn, assistive technology is essential to the realization of rights, as outlined within the UN Convention on the Rights of Persons with Disabilities (CRPD) [6].

Despite the compelling link between assistive technology and human rights realization [7], the World Health Organization (WHO) estimates 9 out of 10 people do not have access to the assistive technology they need, with a higher demand for AT in the Global South. In low- and middle-income countries [8], access to AT is prohibited by high cost and low quality, as well as shortages of skilled personnel within AT production and procurement [9]. In Kenya, 90% of people with disabilities who responded to the national survey for persons with disabilities perceived not having access to assistive products as problematic; however, only 32% of those respondents currently use an assistive product or support service, with the most common products reported as information devices at 20%, followed by mobility aids at 13% [10]. Of the proportion of AT users sampled, nearly half reside in Nairobi, despite the capital city having the lowest disability prevalence (1.1%) of all Kenyan counties measured in the National census [1]. These statistics poignantly highlight the need for increased AT access among people with disabilities in Kenya. As one example, AT has been shown to enable people with disabilities in Kenya to access education and social participation, but access to such technology can be cost-prohibitive in low-income settings [11–13]. Moreover, recent research exploring the experiences of Kenyan wheelchair users points out that even when Kenyans with disabilities have access to AT, stigma related to AT can negatively impact AT uptake [14]. Unfortunately, there is limited data on the uptake, production, and availability of assistive technology within Kenya. It is therefore important to understand the barriers to accessing AT in Kenya, to adequately and appropriately attempt to improve access at a systemic level.

The objective of this research was to describe the assistive technology ecosystem in Kenya through descriptive information about key stakeholder organizations, and a network analysis demonstrating the nature and strength of relationships between organizations. An assistive technology ecosystem is an interconnected community of actors, including government, civil society, and the private sector who work together or in parallel to deliver assistive products and services to the people who need them. Using the network analysis, we aimed to demonstrate the degree to which key stakeholder organizations within the ecosystem did (or did not) interact, which will provide policy makers with data from which to further develop collaborations within the ecosystem.

## Methods

We conducted a mixed methods online survey of assistive technology stakeholder organizations using the Qualtrics platform, an online survey platform which allows for asynchronous and distance-based data collection by respondent organizations. Our mixed methods approach was a partially mixed concurrent equal status design, where the data were quantitative and qualitative data were collected concurrently, and given equal weight in the analyses and reporting [15]. The use of both qualitative and quantitative questions allowed an opportunity to evaluate the nature of the AT ecosystem numerically, in a way which could be easily compared in future work, as well as to understand the nuances of current AT provision using open-ended qualitative questions. Data were collected between March and November 2021. Network analysis was completed with NodeXL software to produce a graphical representation of organizational relationality within the assistive technology network in Kenya. Ethical approval for the study was granted by University College London Research Ethics Service and University of Nairobi.

### Participants

We included participants who were representatives of assistive technology stakeholder organizations including government ministries and agencies, international and local non-governmental organizations, organizations of persons with disabilities, health service providers, and assistive technology innovators and related services. All participants were over the age of 18, participated as a single representative for their organization, and were able to respond to the survey in English. Participants, recruited purposively to represent a range of assistive technology stakeholder organizations, were identified by the research team who have extensive experience working in Kenya in the field of assistive technology. All organizations who have been identified to be engaging in assistive technology provision, service, or disability-related advocacy work were included, and asked to self-identify an appropriate respondent for the survey. Participants were sent a unique link to the survey, with follow-ups to non-respondents at two-week intervals. A member of the research team followed up directly with non-respondents by email or phone on two subsequent occasions.

### Survey content

The online survey consisted of three sections with a total of 17 questions, and was based on a similar survey conducted previously in Malawi to evaluate the AT ecosystem. Following survey development, the survey was piloted with members of the research team in Kenya to identify any potential changes which needed to be made prior to distribution. The first section constituted an informed consent process, where the respondent was provided with information about the survey and use of the data. Proceeding to the second section of the survey was taken to indicate consent. In the second section, we collected organizational demographic data including the type and purpose of the organization, information about clients served (i.e. disability area, age, number per month), and assistive technology products and services provided. Questions regarding assistive products and services provided were linked to the 50 products of the World Health Organization Priority Assistive Product List (APL). The products on the APL were identified through a global consensus process and represent the range of products which should be available through universal health coverage to ensure participation of people with functional limitations in their societies. Each of the questions in the second section used either checkboxes, and open-ended quantitative (e.g. number of employees, number of clients served per month) or qualitative questions (e.g. purpose of organization, disability areas served, challenges in AT provision).

The third section constituted the network analysis, where each respondent was asked to select all of the organizations they were aware of from a pre-populated list of assistive technology stakeholders, and to identify the nature of their organization’s relationship with each of the other respondent organizations over the past twelve months (year) using a defined rating scale. Table 1 outlines the rating scale participants used to rate the nature and strength of the current relationship between their organization and each of the organizations listed on a scale from 1 to 4. This rating scale was also used in a similar network analysis in Malawi [16].

**Table 1.** Description of survey scale used to rate the nature and strength of stakeholder relationships.

	Short Title	Description
1	Awareness	We are aware of the work done by this organization, but our work is entirely independent.
2	Communication	Our organization actively shares information with this organization as we work towards our own goals. We do not currently cooperate or collaborate on any initiatives.
3	Cooperation	Our organization actively shares information, and sometimes has shared activities (less than three times a year). Referral of clients is included in this category.
4	Collaboration	Our organization actively shares information, and frequently has shared activities (more than three times a year). We plan and work together towards shared goals, projects, and initiatives.

Participants were also given the opportunity to suggest organizations which were not listed (open-ended), with whom they had an ongoing relationship in the area of assistive technology, and to rate the nature of those relationships as well. To reduce burden, we asked respondents to rate the relationship only for those organizations which they identified as having awareness of (i.e. if no awareness was indicated, no rating was requested, and a score of 0 was assigned to indicate no relationship).

### Analysis

Quantitative organizational demographic data and information about assistive technology provision from the second section were analyzed as counts and proportions. Qualitative data provided in open ended questions were analyzed using conventional content analysis and summarized for presentation. Conventional content analysis allows thematic analysis of qualitative data where no pre-existing theory exists relevant to the data collected [17]. Codes were derived from the data to represent themes within the data and presented in the results.

A network analysis was conducted using data regarding relationships between organizations (collected from the survey scale in Table 1). NodeXL software was used to produce a graphical representation of the assistive technology network and network metrics for each type of stakeholder. Stakeholders were grouped into organization types to maintain confidentiality of respondents, so no single stakeholder can be identified in the presented results. Network metrics included weighted (by strength of relationship) and non-weighted InDegree metrics, representing the number and strength of inbound relationships (relationships reported by other organizations) and Outdegree metrics, representing the number and strength of outbound relationships (relationships reported by the organization themselves). We also reported on betweenness centrality, a measure of centrality to the network.

## Results

A total of 37 organizations responded to the survey out of 76 initially identified, representing a response rate of 48.7%. Organizational demographic information is presented in Table 2.

**Table 2.** Assistive technology stakeholder organization demographics.

Variable		Number (*n*)	Proportion (%)*
Type of Organization			
	Government Ministry or Agency	5	13.51
	Service Delivery Organization	16	43.24
	Organization of persons with disabilities	10	27.03
	International NGO	8	21.62
	Other**	3	8.11
Disability Areas Served			
	Physical	15	40.54
	Developmental	7	18.92
	Sensory (Hearing/Vision)	11	29.73
	Intellectual	3	8.11
	All	12	32.43
Age of Clients (years)			
	0–4	16	43.24
	5–12	23	62.16
	13–18	25	67.57
	19–50	32	86.49
	50+	21	56.76
Assistive Technology Activities			
	Provide AT directly to clients	20	54.05
	Provide AT-related services	20	54.05
	Provide neither AT nor related services	10	27.03

All totals equals more than 100% as respondents were free to choose more than one category; *Includes charitable trust (*n* = 1) and faith-based organizations (*n* = 2).

Respondents could select more than one option, which enabled the survey to capture organizations which provided both AT and AT-related services directly to clients, as displayed in Table 2 where the total responses (40) differ from the total respondents (37). Conversely, ten respondents indicated their organization provided neither AT nor AT-related services, which could be explained by stakeholders operating higher up within the network, such as policy makers.

### Available assistive products and services

To determine the types of assistive products and services available in Kenya, we asked respondent organizations who indicated that they provide AT directly to clients, to indicate which of the 50 products on the WHO Priority Assistive Product list they provide. Respondents who indicated that they provided AT related services were asked to indicate which products from the AP list they provided service for. Respondents from organizations which do not provide any AT or AT related services were not prompted to complete this section of the data collection.

Table 3 shows the number of organizations providing specific AP directly or providing related services for. Of the 50 products on the WHO APL, only one product (fall detectors) was not selected by any organizations for providing the product itself, nor related services. Eleven additional APs (alarm signalers, closed captioning displays, gesture to voice technology, hearing loops/FM systems, keyboard and mouse emulation software, personal digital assistants, personal emergency alarm systems, pill organizers, simplified mobile phones, time management products, and video communication devices) were not provided directly by any of the respondent organizations, however did have related services provided by at least one of the respondents. Four organizations identified providing additional products which were not on the WHO APL including easy to read information, sitting and standing aids, parallel bars, sleep positioning equipment, and sign language interpretation technology.

**Table 3.** Assistive products and services provided by assistive product.

	# of Organizations Providing …	
Assistive Product	Product	Service*
Alarm signalers with light/sound/vibration	0	2
Audioplayers with DAISY capability	1	2
Braille displays (note takers)	3	5
Braille writing equipment/braillers	3	6
Canes/sticks	6	8
Chairs for shower/bath/toilet	6	8
Closed captioning displays	0	2
Club foot braces	5	6
Communication boards/books/cards	3	8
Communication software	3	6
Crutches, axillary/elbow	11	8
Deafblind communicators	1	3
Fall detectors	0	0
Gesture to voice technology	0	1
Global positioning system (GPS) locators	1	1
Handrails/grab bars	2	6
Hearing aids (digital) and batteries	4	5
Hearing loops/FM systems	0	1
Incontinence products, absorbent	2	2
Keyboard and mouse emulation software	0	2
Magnifiers, digital hand-held	2	2
Magnifiers, optical	3	3
Orthoses, lower limb	6	6
Orthoses, spinal	3	4
Orthoses, upper limb	4	3
Personal digital assistant (PDA)	0	1
Personal emergency alarm systems	0	1
Pill organizers	0	1
Pressure relief cushions	8	5
Pressure relief mattresses	2	3
Prostheses, lower limb	6	5
Ramps, portable	4	5
Recorders	1	2
Rollators	1	2
Screen readers	1	2
Simplified mobile phones	0	1
Spectacles; low vision, short distance, long distance, filters, and protection	2	3
Standing frames, adjustable	5	4
Therapeutic footwear; diabetic, neuropathic, orthopaedic	2	2
Time management products	0	1
Travel aids, portable	1	1
Tricycles	9	10
Video communication devices	0	2
Walking frames/walkers	7	9
Watches, talking/touching	2	2
Wheelchairs, manual for active use	11	10
Wheelchairs, manual assistant controlled	7	6
Wheelchairs, manual with postural support	9	7
Wheelchairs, electrically powered	3	1
White Canes	5	3
Other	4	3

*Service indicates the education, training and fitting of APs listed.

For those organizations providing products directly to clients (*n* = 20), 14 (70%) indicated they purchase the products, 10 (50%) indicated they build the products themselves, and 13 (65%) receive and distribute donated products. Of those same organizations, 6 (30%) indicated clients pay a portion of the cost of the device, 6 (30%) indicated clients pay a fixed cost, and 13 (65%) indicated clients receive their devices for free.

### Challenges delivering assistive products and services

Respondents indicated they experience several challenges related to obtaining, producing, or distributing assistive products. These have been grouped into five thematic areas: AP supply, AP cost, AP quality, manufacturing challenges, and service challenges.

Respondents indicated a number of specific challenges with *AP supply. One respondent representing an International NGO shared that i*n the face of ‘overwhelming demand,’ where demand for products is often greater than available supply, organizations identified a lack of appropriate products, inconsistent supply of donated products, and delivery delays including challenges with ‘clearance at the port.’

Where there is available supply, respondents also described challenges with *AP cost*. While several respondents noted the cost of products themselves, they also noted systemic issues. In particular, one respondent representing a Service Delivery Organization pointed out the high cost of device transportation and importation: ‘parts attract VAT [import tax] thus becoming very expensive to the user to afford.’

Challenges were also identified with *AP Quality*, where several organizations who manufacture their own products identified challenges with maintaining low cost without compromising on quality, as well as poor quality of imported parts. Furthermore, those organizations relying on donations noted inconsistency in the quality of donated products.

Respondent organizations who provide services to clients also noted a number of specific *Service Challenges*. They described poor referral services, difficulty reaching rural AP users, in part due to a lack of funds, and a lack of follow-up due to a lack of accessible transport for service users.

Those who manufacture APs locally also described several *Manufacturing Challenges*. They described difficulty obtaining appropriate materials and manufacturing equipment, inadequacy of locally available manufacturing materials, challenges relying on a supply of donated materials, and difficulty obtaining adequate skilled labour to build APs.

### Network analysis

**Figure 1.**
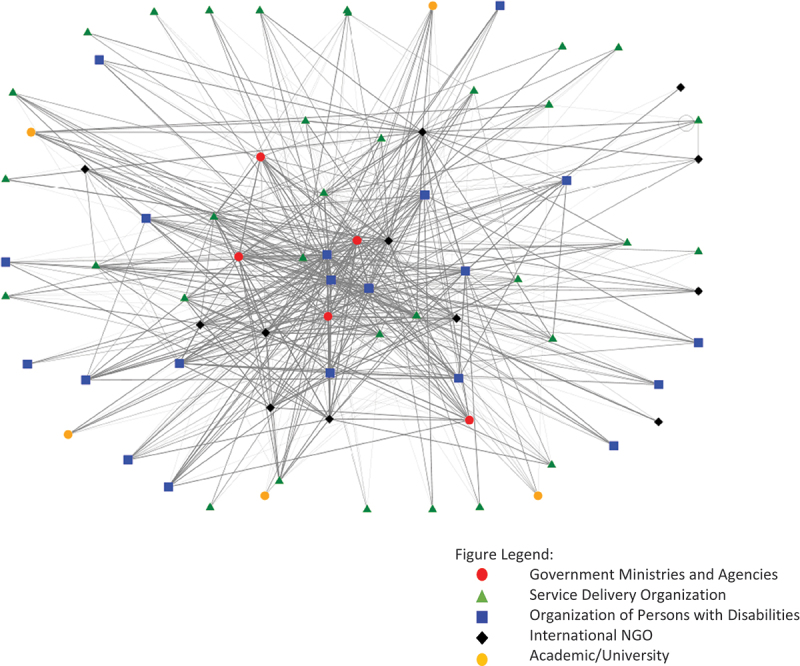
Kenyan assistive technology network.

The network analysis shows a distributed network with a range of actors participating in the network, including government ministries and agencies, service delivery organizations including healthcare facilities, organizations of persons with disabilities, international NGOs, and innovators. The network is represented graphically in Figure 1 and shows ministries and International NGOs as being quite central to the network, with service delivery organizations and organizations of persons with disabilities being more distributed. Innovators are less connected to the network than the remaining types of organizations. In Figure 1, distinct types of organizations are depicted by differing shape and colour of icon. Lines between organizations depict directionality (i.e. from the respondent organization to the other) and strength of the relationship (stronger relationships show thicker lines). In cases where an organization did not respond to the survey, lines joining them to other organizations are unidirectional (i.e. from a respondent organization to the non-respondent organization).

Network metrics were calculated to quantify the nature and strength of the network and relationships between stakeholders. To maintain confidentiality, these metrics have been presented by organization type rather than by individual organization. These metrics can be seen in Table 4. Innovation training organizations, organizations which provide support and training to innovators who are developing new products and services, are included in the table but are not included in the demographic information. We did not have any responses from these organizations, however they were reported as collaborators by respondents, therefore are included in the table with only two metrics – indegree, and weighted indegree.

**Table 4.** Network metrics by organization type.

	Means by Organization Type					
Organization Type	Indegree	Outdegree	Weighted Indegree*		Weighted Outdegree*	Betweenness Centrality*
Government Ministry or Agency	19.80	22.40		59.00	55.20	157.52
Organization of Persons with Disabilities	11.50	17.73		29.90	51.00	109.35
Service Delivery Organization	7.27	15.29		16.12	29.21	58.56
International NGO	12.00	11.08		21.71	27.67	58.86
Innovation Training Organization	8.40	–		20.20	–	–

*Means for Outdegree, Weighted Outdegree, and Betweenness Centrality include only respondent organizations.

The metrics of indegree and weighted indegree represent all organizations listed in the survey, and not only respondents, likely contributing to the differences between the indegree and outdegree scores. Overall, Government Ministries and Agencies (represented by the red circular nodes in Figure 1) have the highest betweenness centrality and are clustered closer to the center of the Network, followed by Organizations of Persons with Disabilities. A higher betweenness centrality score suggests these organizational types play a lead role in connecting organizations to one another, and collaborating across the sector, which is visually depicted in Figure 1 through their central location.

## Discussion

This is the first study to systematically explore the assistive technology ecosystem in Kenya, including the products and services available, as well as the relationships between key stakeholder organizations. Through mapping how key organizations are currently interacting (or not interacting) with each other, the findings demonstrate the interconnectivity of the AT ecosystem in Kenya through the relationality of its stakeholders. The network analysis highlights existing relationships as primarily rooted in awareness and communication, rather than cross-collaboration among organizations. While the data suggests Government Ministries and Agencies, as well Organizations of persons with disabilities, are situated centrally within the network and facilitate the interconnectivity of organizations, it appears those connections do not largely lead to collaboration. Meanwhile, the service delivery organizations and International NGOs have lower ‘betweenness centrality’ scores, suggesting their positioning within the ecosystem is more peripheral. Both service delivery organizations and International NGOs had smaller weighted indegree and outdegree scores, indicating that despite their existing relationships generating inbound and outbound interactions, the overall strength of those relationships remained weaker and thus less likely to result in collaboration. Finally, innovation training groups are yet to be integrated into the network at all.

The results from this study are similar to the findings from a similar study conducted in Malawi, which found a highly distributed network, with no single organization identified as most central to the network [16]. Similarly to Kenya, international NGOs in Malawi were less central to the network than those leading from within [16].

Strengthening existing relationships across the sector may lead to more collaborative inbound and outbound interactions rooted in reciprocal engagement, versus interactions centered on bilateral awareness or communication. Rather than organizations working in silos, a collaborative approach within the AT ecosystem could enable organizations to leverage the expertise, resources, and existing programming of other groups within the sector who have similar missions, values, goals, and beneficiaries. Fostering stronger relationships among organizations within the network, through a systems thinking approach, could also enable collaboration in navigating shared challenges, better equipping them to challenge systemic barriers to AT access for people with disabilities in Kenya [18]. In particular, it is worth mentioning the potential of Government Ministries to facilitate stronger organizational collaboration due to their central positionality within the network (as highlighted in Figure 1). For example, health systems strengthening literature in low and middle income countries suggests the need to improve overall organizational capacity as well as institutions, which could be done in partnership with other organizations to raise the status of the ecosystem as a whole [19]. One way the Government Ministries could enhance organizational capacity and improve interconnectivity of the AT network in Kenya is to develop appropriate policies on AT in Kenya, support local innovators creating AT and control importation taxes on AT and related parts or materials.

Additionally, building new relationships within the sector and increasing the interconnectivity of the ecosystem could also promote collaboration among AT stakeholders within the network. Increasing the betweenness centrality scores of service delivery organizations and International NGOs would suggest an enhanced ability to form connections more independently, rather than relying on Government Ministries and Agencies or Organizations of persons with disabilities to connect them with other groups. Moreover, integrating innovation training groups within the sector could not only increase the breadth of relationships within the network, but help to bridge existing gaps in AT access.

For example, respondents indicated several challenges with the delivery of assistive products and services, such as high costs related to the transportation and importation of AT parts and products. The introduction of new players within the ecosystem, could inevitably offer novel and innovative strategies to address AT access, while also challenging the notion of where innovation takes place [20], or rather who holds the power to innovate within the AT sector. With the example of high transportation and importation fees in mind, collaboration with innovation groups within the ecosystem could modify the AT procurement chain through offering high-quality domestic production, circumventing reliance on internationally donated products and expensive importation fees [21].

### Recommendations for Kenya and countries with similar ecosystems

Understanding the distribution of organizations, the strength of their relationships and subsequently the nature of their interactions, can ultimately offer important insights to aid the promotion of AT access within context. We therefore encourage future researchers and practitioners to build upon this work to implement collaboration within AT ecosystems as a mechanism to address AT access, particularly within low- and middle-income countries such as Kenya. This project was limited by a low response rate, as well as zero responses from Innovation training organizations which provide support and training to innovators developing new AT related products and services. It is therefore recommended that future researchers and stakeholders within AT networks consider these factors when taking up and building upon this work, such as future studies which focus on AT innovation in low-income settings.

Based on our findings, the following actions may be considered to improve the strength of the assistive technology ecosystem in Kenya and in other countries with similar challenges.

1. Develop mechanisms for meaningful collaboration between key stakeholders, across differing stakeholder groups; and
2. Identify organizations innovating within the assistive technology ecosystem and provide the necessary support for their integration into the broader assistive technology service delivery ecosystem; and
3. Invest in capacity building across the assistive technology sector to improve access to technologies for persons with disabilities; and
4. Ensure meaningful engagement of organizations which represent persons with disabilities in policy planning and implementation.

## Conclusions

This is the first study to explore the nature of the assistive technology ecosystem, and the relationships between key stakeholders in Kenya. The assistive technology network in Kenya is distributed, with Government Ministries and Agencies and Organizations of persons with disabilities central to the network. The strength of relationships is concentrated on awareness and communication, with fewer organizations actively collaborating. Innovation training organizations are not yet well integrated into the network. Improving access to assistive technology in Kenya will benefit from greater collaboration amongst all assistive technology stakeholders. Understanding the AT ecosystem in Kenya may provide insights into access to assistive technology for persons with disabilities in similar resourced environments facing similar challenges.

## Paper context

There has been limited research on the assistive technology ecosystem in Kenya, primarily limited to exploration of the development and application of specific technologies in the Kenyan context. This research explores the current assistive technology landscape to provide a baseline understanding of the conditions for assistive technology implementation and policy and systems change. Understanding the nature and strength of relationships within the assistive technology ecosystems may be used to further strengthen the field.
